# A Novel Optical Instrument for Measuring Mass Concentration and Particle Size in Real Time

**DOI:** 10.3390/s23073616

**Published:** 2023-03-30

**Authors:** Jingxiu Zhang, Zhiwei Zhang, Longfei Hou, Weihu Zhou

**Affiliations:** 1 School of Aeronautic Science and Engineering, Beihang University, Beijing 100191, China; 2 School of Precision Instrument and Opto-Electronics Engineering, Tianjin University, Tianjin 300072, China; 3 College of Automation Engineering, Nanjing University of Aeronautics and Astronautics, Nanjing 210016, China; 4 University of Chinese Academy of Sciences, Beijing 100049, China; 5 Institute of Microelectronics of the Chinese Academy of Sciences, Beijing 100029, China

**Keywords:** particle size, aerodynamic particle size, anti-overlap algorithm, particulate matter

## Abstract

Particle mass and particulate size are two important parameters used to characterize the aerosol. Currently, there are a few methods for measuring particle mass concentration and particle size. However, the existing methods have their own shortcomings. In this article, we describe a novel laser scattering instrument that measures mass concentration and particle size in real time over a wide concentration range. This instrument combines laser scattering and time-of-flight aerodynamics in one optical device. There are two innovations in this paper: (1) Two APD detectors are used to receive scattered light. One receives forward-scattered light and the other receives side-scattered light. The advantage is that the sensitivity of the detector is increased greatly, and the ratio of forward and side scattering is used to further obtain the size and shape information of the particles. (2) In order to measure the high concentrations of aerosol, a high-speed ADC and FPGA is combined to achieve an anti-overlap algorithm objective. It has been verified by experiments that the anti-overlapping algorithm can effectively improve the applicability of the aerodynamic particle size spectrometer under high concentration conditions.

## 1. Introduction

Industry’s rapid development in recent years has greatly contributed to China’s economic progress, but it has also caused numerous air quality problems. Haze weather frequently occurs as industrial pollution intensifies. Among all air pollutants, particulate matter is the most serious and has the greatest effect on human health. Air pollution control has become a public concern to ensure a reasonable development of industry [1]. Hence, the detection of air quality is particularly important. The monitoring and analysis of the concentration of particulate matter in the atmosphere becomes a prerequisite for environmental governance.

Particle size and mass are two important parameters used to characterize an aerosol. The atmospheric particulate concentration is a basic parameter for characterizing the spatial distribution of the particulate matter, which is generally expressed by mass concentration and quantity concentration [2]. According to the aerodynamic diameter, atmospheric particulate matter can be divided into four levels: (1) total suspended particulate matter (TSP) with a diameter of less than 100 μm; (2) respirable particulate matter with a diameter of less than 10 microns (PM10); (3) a diameter less than 2.5 μm fine particles (PM2.5), which can be suspended in the atmosphere for a long time, bringing an important impact on air quality and visibility; (4) ultrafine particles with a diameter less than 1 micron (PM1), which can easily enter various tissues of the human body by respiratory system. PM2.5 and PM1 have a small particle size and strong chemical activity, and are prone to carry toxic and harmful substances (such as heavy metals, viruses, microorganisms, etc.). They stay in the atmosphere for a long time and move a long distance. Therefore, PM1 and PM2.5 have greater impacts on human health and atmospheric environmental quality [3,4,5].

The standards for ambient air quality (PM1, PM2.5, and PM10), exposure assessment for inhalable particulates, and FDA guidelines for pharmaceutical aerosol characterization are based on mass and aerodynamic size of the particles. Most of this aerosol characterization will benefit from the real-time measurement of mass-weighted aerodynamic size distributions, which will significantly reduce the time required to characterize aerosols and provide higher resolution particle size data. The demand for concentration statistics over different particle sizes is increasing with environmental requirements.

At present, there are some common methods for measuring particle mass concentration and particle size: the β-ray absorption method [6,7], the QCM(Quartz crystal microbalance) [8,9], the charge transfer method [10], the optical scattering method (laser scattering method) [11,12,13], and the time-of-flight aerodynamic method [14,15,16]. The β-ray absorption method has good mass sensitivity [6,17]; however, the disadvantage of the β-ray absorption method is that it is time consuming and expensive [17]. Additionally, the sampling paper tape inside must be replaced manually. The β-ray absorption method and QCM method have good mass sensitivity, but are unable to measure particle size without a size selective inlet. The charge transfer method is the most used for nanoparticles.

The optical scattering method is widely used for measuring particle size distribution in real time [17,18]. Optical measurements of particle velocities are widely used to study particle dynamics and gas flows. The measurement of particle size is used in a variety of fields, including pollution and contamination monitoring, respirable particle mass monitoring, and spray nozzle performance monitoring. Optical scattering has the following advantages: (1) accurate particle counting when the concentration is low; (2) good signal-to-noise ratios when the particles are larger (e.g., >100 μm); and (3) low cost. There are several disadvantages to the optical method: (1) If particle density is unknown, optical size does not equal geometric size since it depends on particle shape and refractive index; this error is exacerbated when particle size distribution is converted to mass concentration. (2) Particle concentrations will be underestimated because multiple particles are present at the same time in the measure volume, causing coincidence errors. Due to these reasons, optical scattering methods are mostly used in clean environments [17].

Time-of-flight velocimetry, also called transit time, two-spot, or two-focus velocimetry, is the most common technique for measuring velocity. Using this method, two beams of light radiation (laser radiation) are directed through a volume of particles. Two pulse signals will be generated when a particle passes through both beams. In order to measure particle velocity over a known distance, a timing signal is initiated contemporaneously with the first pulse and terminated concurrently with the second pulse. The advantages of time-of-flight aerodynamic method is that it is less dependent on the particle refractive index and density than optical scattering method. Good agreement between the time-of-flight aerodynamics and direct mass measurements has been reported [19]. However, a commercial instrument that uses time-of-flight aerodynamics (for example the APS3321 from TSI) cannot measure high concentrations [20], mainly due to the defects of its signal processing circuit and its data processing algorithm [21].

According to these methods and instrument characteristics, Table 1 briefly compares the advantages and disadvantages of the methods for measuring particle concentration and particle size.

In this paper, we design a novel laser scattering instrument that measures mass concentration and particle size in real time over a wide concentration range. The novelty of this instrument is that it combines laser scattering and time-of-flight aerodynamic in one optical device. We use two APDs in order to increase the sensitivity and obtain more information of the particles. In order to measure higher particle concentrations, we use digital acquisition technology for implementing anti-overlapping algorithms, which solve the problem of overlapping particles interfering with each other in high concentrations.

## 2. Principle of Measurement

### 2.1. Instrument Description

**Figure 1:**
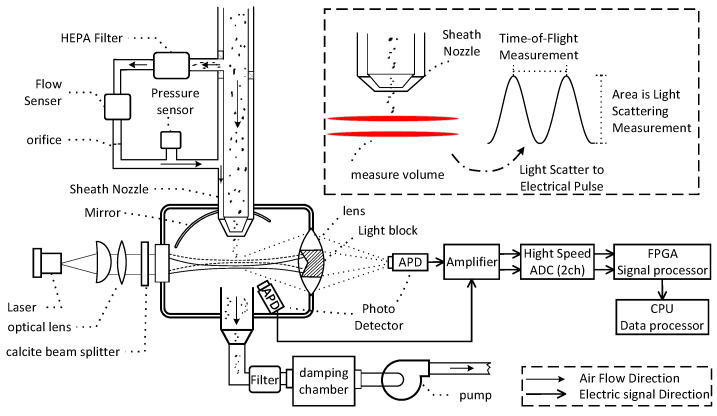
Schematic diagram of the novel instrument. The particle beam, laser beam, and the axis of the mirror are orthogonal to each other.

The novel instrument is shown in Figure 1. Using a pump with a damping chamber at a total flow rate of 5 L/min, clean air and aerosol (air with particulates) are drawn into the optical chamber through the sheath nozzle in a continuous stream. The air is filtered to remove particulates and becomes clean air through a HEPA filter. The clean air is then drawn back into the optical chamber around the inlet nozzle as sheath flow to reduce particles and protect the optics from particle contamination. The remaining 1 L/min of air with particulate matter through the inlet of the sheath nozzle enters the optical chamber. As particles pass through the measurement volume, they are illuminated by a parallel laser beam with a wavelength of 635 nm. The avalanche photo detector (APD) captures side-scattered light in the scattering angle of 30° to 120° using a spherical mirror. A lens focuses the forward-scattered light onto the second APD. Signals from the APD are converted into digital form by high-speed ADCs and processed by FPGAs. In order to maintain pressurized drop balance in the two flow paths, an orifice is used to maintain the aerosol-to-sheath flow ratio. The HEPA filters are used to filter dust from the sheath flow before affecting the flow rate. This damping chamber is used to reduce the air jitter that is caused by the pump, thereby maintaining a steady flow rate at the nozzle.

**Figure 2:**
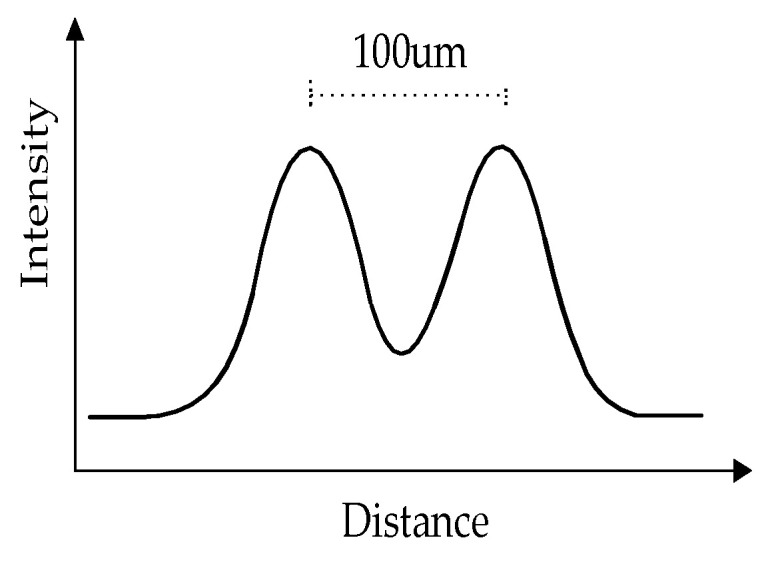
Light intensity profile over measurement volume.

**Figure 3:**
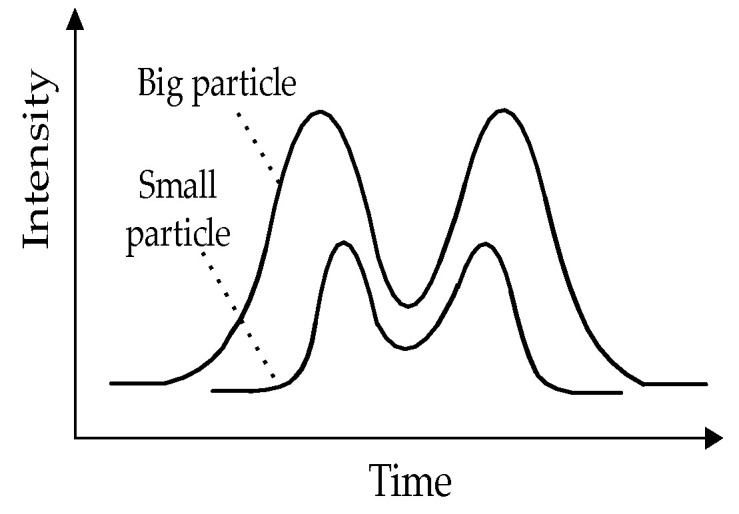
Relationship among particle size, TOF, and intensity.

The laser is shaped by an optical lens, then split into two beams by calcite spaced 100 μm apart. Each beam is 1 mm wide and 40 μm thick. Figure 2 shows the light intensity over the measuring volume, double-peaked from top to bottom. A reduced pressure is created and maintained by the vacuum pump, so that the clean gas at the nozzle is ejected at the same speed. With the same pressure, different particles will move through the measuring volume from top to bottom at different rates. Larger or heavier particles scatter more lights and move over the measuring volume more slowly. This affects the relationship among particle size, time of flight (TOF) and intensity, as seen in Figure 3. It can be used to estimate the aerodynamic particle size of particles ranging from PM0.3 to PM20 based on this property.

### 2.2. Intensity Ratio of Backward Scattering to Forward Scattering

**Figure 4:**
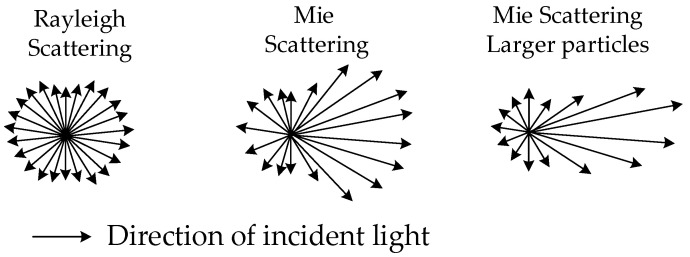
Relationship between size and scattered light intensity distribution.

The laser scattering method principle calculates particle size distribution from the scattered light intensity distribution (scattering pattern). When the laser irradiates the particles, if the particle size exceeds the wavelength of the laser, the particles will scatter the light in the same direction as the laser light (forward scattering). If the particle size is approximately equal to or smaller than the wavelength of the light, the scattered light increases in perpendicular directions (lateral) and in directions backward (backward scattering) [22,23,24,25,26], show as Figure 4.

The device has two photo detectors, one of which is used to collect sideward and backward-scattered light (scattering angles between 30° and 120°), the other to collect forward-scattered light. It offers two benefits: (1) The signal-to-noise ratio is increased by combining the signal values from the two detectors. (2) As the particle diameter decreases, the ratio of backward scattering plus sideward scattering to forward scattering increases. As a result, this ratio can be utilized to increase the precision of small particle measurements.

### 2.3. Time of Flight

The Bernoulli’s equation is a basic equation in fluid dynamics [27]. According to Bernoulli’s equation (Equation (1)), when the pressure difference between the inside and outside of the nozzle of the instrument is 15 kPa, the air spray velocity of the sheath nozzle will be 150 m/s.

(1) $$P_{1}+\frac{1}{2}\rho v_{1}^{2}=P_{2}+\frac{1}{2}\rho v_{2}^{2}$$

The velocity of particles relative to the air in the nozzle can reach approximately one third the speed of sound (Table 2) [28].

The Reynolds number is the ratio of inertial forces to viscous forces within a fluid The larger the particle’s Reynolds number, the slower the particles move in the fluid [27,29].

As shown in Figure 1, the distance between two parallel beams is 100 μm; based on Table 1, PM0.5-PM20 particles have a flight time (Time-of-Flight) ranging approximately from 700 to 3500 nanoseconds.

### 2.4. Digital Signal Processing and Anti-Overlap Algorithm

**Figure 5:**
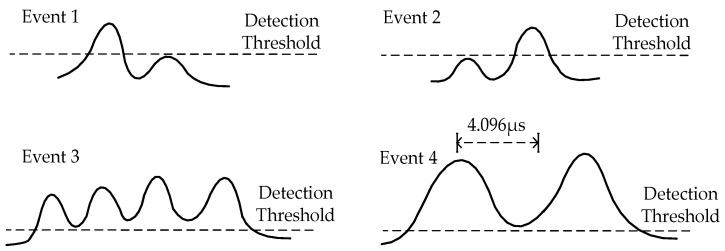
Complicate events which analog circuits cannot handle. In events 1 and 2, when the signal from a small particle cannot remain above the threshold, only one crest is detected, and no time-of-flight measurements are taken. In the case of event 3, although the signal remains above the threshold, three or more crests are detected as a result of coincidence. Such events are logged, but concentration and flight time are not recorded. Event 4 is outside the timer’s maximum range, and in this case, the signal re-mains above the threshold until it moves outside the timer’s range, and only one crest is observed. Event 4 is typically caused by large or recirculating particles, and in this case, the event will be logged, but no time-of-flight is recorded.

This paper proposes the use of high-speed ADC for photoelectric signal acquisition, and FPGA for signal processing to replace previous analog circuits [21]. The advantage is that digital signal processing techniques is capable of processing complex waveforms. With the help of ADC and FPGA digital circuitry, it is possible to process waveforms shown in Figure 5, which are impossible for analog circuits [20,30].

**Figure 6:**
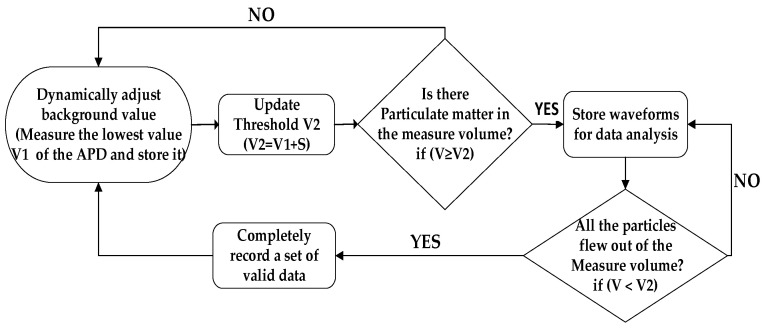
Processing flow of signal acquisition and storage.

In analog circuits, the threshold is set to a fixed value. The background signal output by the APD fluctuates widely due to temperature, gain, etc. If the threshold is lowered, the instrument will not operate correctly. The digital signal processing techniques proposed in this paper can be used to dynamically detect the APD’s background value so that the threshold value also fluctuates with the fluctuation of the background signal, causing waveforms such as events 1 and 2 to be processed accurately. For detailed processing flow, please refer to Figure 6, in which V is the voltage value of the APD output; V1 is the lowest value detected in the current loop; and the threshold V2 is equal to the manually set value S plus V1. Event 4 is also easily handled using the processing flow shown in Figure 6.

**Figure 7:**
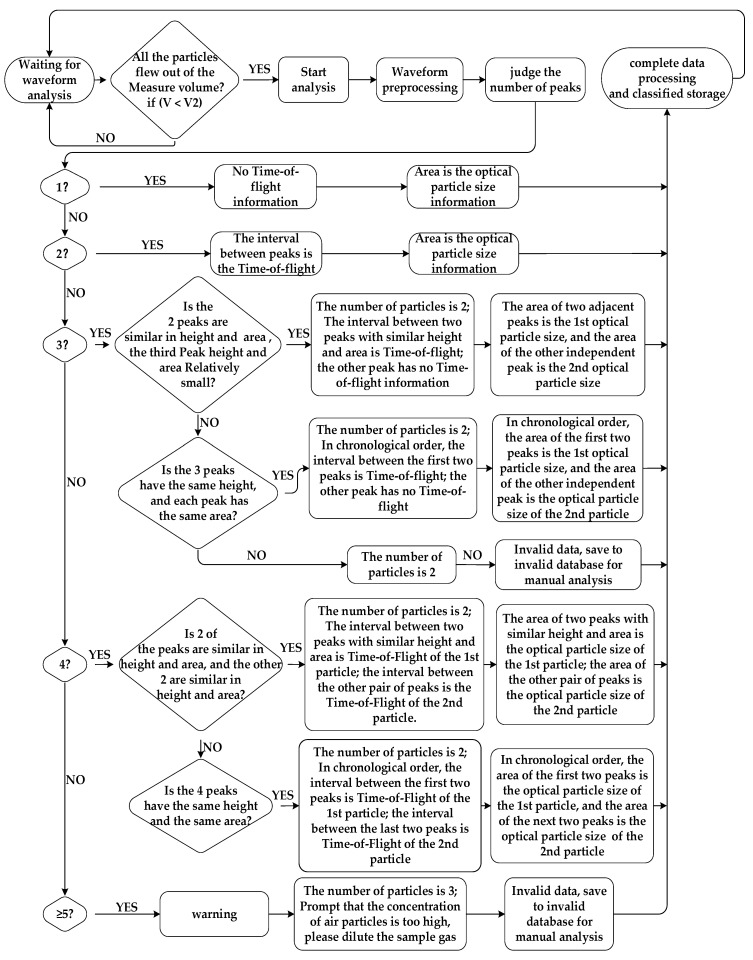
Processing flow for anti-overlap algorithm.

It is easy to process the waveforms of event 3 using digital processing techniques. Event 3 is called particle overlap. There are many ways to handle event 3. This paper proposed an anti-overlap algorithm, which can be achieved using the data processing flow shown in Figure 7.

The system works in an uninterrupted and real-time mode. As soon as a particle flies out of the measuring volume, the data is analyzed. To ensure that no data are lost, the algorithm for particulate matter needs to be completed before the next particulate matter flies out of the measurement volume. The time for particles to fly through the measurement volume is approximately 600 ns–4000 ns, which means that the algorithm needs to be completed within 600 ns. Therefore, an FPGA must be used to implement the algorithm.

## 3. Results and Discussion

Some experiments have been conducted to test the instrument’s performance using TSI Company’s 3410U and CMAG 3475 aerosol generators. These generators generate particles of varying sizes for experimentation, and the relationship between particle concentration and scattered light intensity is observed [31]. The 3410U is an aerosol generator, which produces aerosols of different sizes from 0.5 μm to 100 μm. CMAG 3475 aerosol generator with Sinclair–Lamer condensation technology produces aerosols of varying sizes (0.5 mm to 8 mm) by controlling temperature and airflow. CMAG 3475 produces particulates in quantities per unit volume.

The specific experiments are as follows:

(1) A calibration experiment for TOF of Aerodynamic Particles was conducted in which 3410U was used to generate 11 different standards of particulate matter. Measure the time-of-flight of 11 different diameters of standard particles and use these data to calibrate the instrument. By calibrating the time-of-flight of 10 different diameters of standard particles, the instrument can measure the time-of-flight of similar particles in the future.
(2) Experiment for “scattered light intensity of p”. After TOF calibration in experiment 1, several different diameters of standard particles are measured again. The instrument performance is analyzed by analyzing the standard deviation of the measured TOF data and light scattering intensity data. The results of the analysis can be used to determine how well the instrument is functioning, and whether or not any adjustments need to be made. The smaller the standard deviation, the better the instrument’s performance.
(3) Experiment for “work in high concentrations”. In this experiment, the CMAG 3475 was used to generate standard particulate matter. This was performed to test the accuracy of the instrument in measuring the number concentration at different concentrations by controlling the change in the number concentration per unit volume. The CMAG 3475 was chosen because it is capable of producing a range of different concentrations of particulate matter while also providing a constant number concentration per unit volume. This allowed for a more accurate test of the instrument’s accuracy in measuring the number concentration of particulate matter at different concentrations.
(4) Experiment for ambient aerosols. In this experiment, a parallel comparison experiment with the particle analyzer of the β-ray principle was performed at the same location. The purpose of the experiment is to evaluate the accuracy of the analyzer by comparing the results of the particles measured by β-ray.

### 3.1. Calibration Experiment for TOF of Aerodynamic Particle

**Figure 8:**
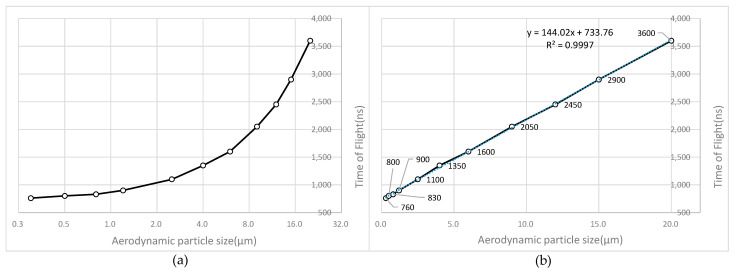
TOF of aerodynamic particle size of PM0.3-PM20. (**a**) A logarithmic base of 2 is used for the abscissa to facilitate viewing the values. (**b**) In order to view linearity comfortably, the abscissa is normal.

In theory, there exists a precise relationship between aerodynamic size and velocity. However, the actual system has some (albeit minor) variations. A series of tests using single spheres of uniform sizes is highly recommended to calibrate the system. Based on these tests, the empirical relationship between TOF measurements and aerodynamic particle size will be established. Figure 8 is a calibration curve using 11 different standards of particulate matter.

**Figure 9:**
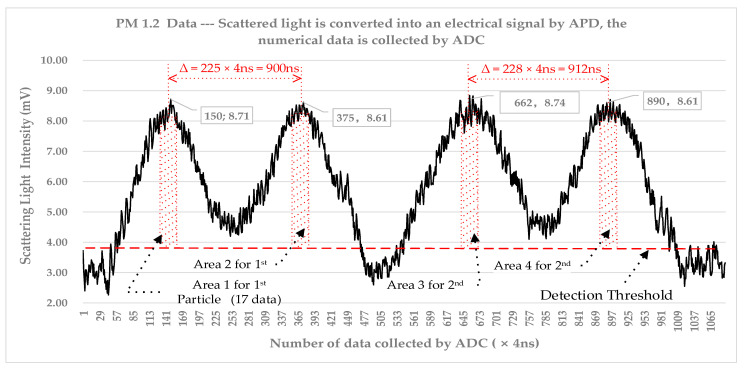
TOF and Scattering Light Intensity of two PM1.2 particles; TOF=∆=Numbers ×4 ns.

Time-of-flight data for 11 different standard particle diameters were obtained, and Figure 8 illustrates the results. The time-of-flight of particles 0.3–20 μm is approximately 700 ns to 4000 ns. In Figure 8b, it is evident that the particle size is linearly proportional to time-of-flight. Figure 9 shows the data collected by ADC when calibrating the instrument with PM1.2.

As shown in Figure 9, the TOF of PM1.2 is around 900 ns. In order to normalize the data, for each particle with TOF, the scattering light intensity value is taken from the highest point on each peak to take its adjacent 16 points, and there are 34 points in total for the 2 peaks. The scattering light particle size of the first PM1.2 is area 1 plus area 2, and the scattering light particle size of the second PM1.2 is area 3 plus area 4.

In order to obtain time-of-flight data for 0.3 μm standard particles, one APD is utilized to receive forward-scattered light and the other APD for side-scattered light; these two APD’s signals are then summed for the same moment of data to increase the signal-to-noise ratio. By using two APDs, the system can measure the time of flight of particles with much higher accuracy and resolution than if only one APD was used. The two APDs also allow for measurements of both forward and side-scattered light, which gives a better overall picture of the particles’ behavior.

### 3.2. Scattered Light Intensity of Particle

**Figure 10:**
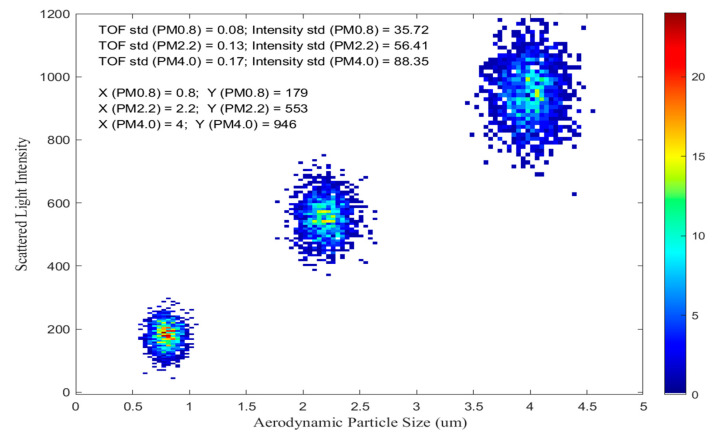
Scattered Light Intensity of PM0.8\PM2.2\PM4.

After time-of-flight calibration, which means that the time of flight corresponds to the particle diameter, samples of standard particles (PM0.8, PM2.2, PM4) were tested with two objectives: one for analyzing the standard deviation of TOF, and the other for analyzing the light intensity of the forward and side-scattered light of the particles. For different diameters of particulate matter, the ratio of forward scattering to side scattering is obtained. To analyze instrument performance, 1000 FWHM data were collected for each particle sample (PM0.8, PM2.2, and PM4.0). Its data heatmap is shown in Figure 10.

Analyzing the data in Figure 10 and Table 3, it is found that the standard deviation of TOF increases with particle size. In addition, the standard deviation of scattered light intensity increases with particle size. The standard deviation of TOF for the same particles is much smaller than the standard deviation of light scattering. This indicates that the TOF measurement is much more precise and accurate than light scattering measurement when it comes to determining the size of particles. This is because the TOF measurement measures the time it takes for a particle to travel a certain distance, so any change in particle size would lead to a change in the time of flight. However, the light scattering measurement is less accurate because it measures the amount of light reflected by the particles, and the amount of light reflected does not necessarily depend on the size of the particle. Therefore, the TOF measurement is more reliable for measuring particle size.

According to the data in Table 3, scattering light intensity increases as particle size increases. The ratio of forward scattering light to backward scattering light increases as particle size increases. This is because, in accordance with Mie scattering theory, the larger the particle’s diameter, the stronger the forward scattering light relative to the side-scattering light. This is due to the fact that larger particles have a larger cross-sectional area, which increases the amount of light that is scattered in the forward direction. At the same time, the size of the particles also increases the probability of light being scattered in the backward direction, resulting in a higher ratio of forward to backward scattering light.

### 3.3. Work in High Concentrations

**Figure 11:**
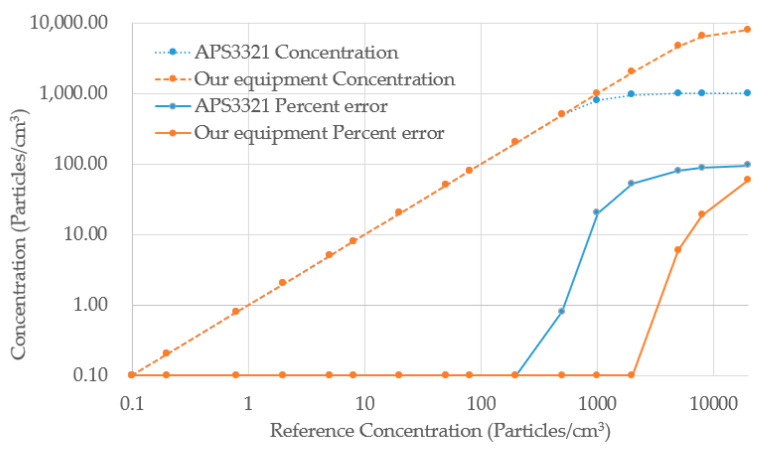
Concentration linearity and percent error of our equipment vs. APS 3321.

A particle generator CMAG 3475 is used to generate PM2.2 in different numbers of concentrations from 0.1 to 10,000 particles/cm^3^. At these different concentrations, our instrument was compared with the TSI 3321. The concentration data curves obtained by these two analyzers are shown in Figure 11.

When the concentration is below 800 particles/cm^3^, both instruments exhibit very good linearity and accuracy. The linearity and accuracy of the APS3321 deteriorates when the concentration of generated particulate matter exceeds 1000 particles/cm^3^. The linearity and accuracy of our equipment is excellent up to a concentration of 8000 units/cm^3^. Our instrument measures maximum particle concentrations of 8000 particles/cm^3^, which is much higher than the 1000 particles/cm^3^ measured by APS3321 [32]. Due to the overlap of particles, the analog circuitry used in the APS3321 is unable to process complex signals caused by high concentrations. In contrast, our instrument uses an ADC to acquire the signal and anti-overlap algorithms implemented in a FPGA to increase linearity and accuracy at high concentrations. This means that our instrument is capable of accurately measuring higher particle concentrations than the APS3321, which is limited by its analog circuitry and the complexity of signals caused by high concentrations. Additionally, our instrument uses an ADC and anti-overlap algorithms to further improve accuracy and linearity. However, there are some drawbacks to this approach. One is that it can be more expensive to produce an instrument with digital circuitry. Another is that the digital approach can be more complex and difficult to troubleshoot than an analog approach.

### 3.4. Experiment for Ambient Aerosols

In this experiment, we compare the data obtained by the β-ray equipment in the standard air station. Through analyzing the data, it can be seen that the PM2.5 and PM10 data obtained by our instrument (TOF) are consistent with the data of β-ray (Thermo-Fisher Model 5014i) made by Thermo-Fisher Environmental Instrument, USA.

**Figure 12:**
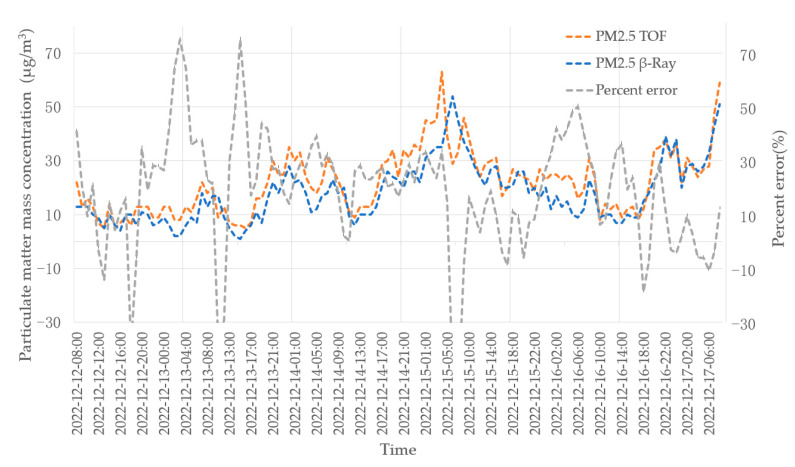
Actual measured concentration of PM2.5.

**Figure 13:**
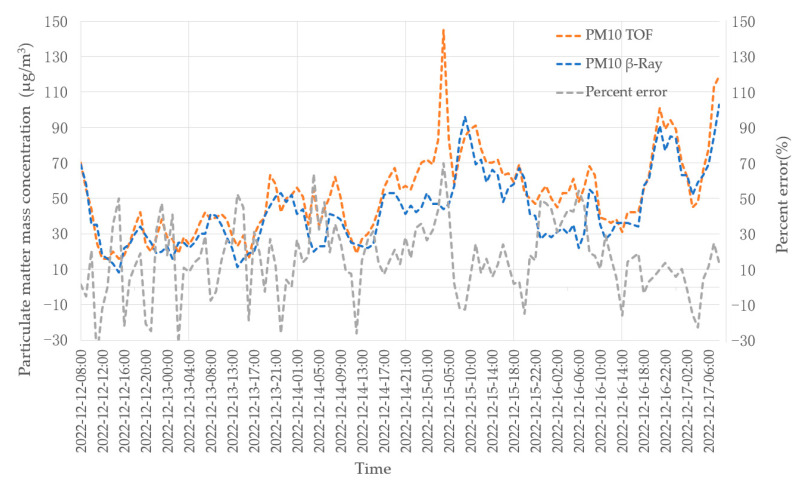
Actual measured concentration of PM10.

Using the method proposed by Thomas M. Peters [16], the number concentration measured by the aerodynamic particle size spectrometer was converted into a mass concentration for comparison with the data of β-ray method. For each TOF channel, the differential mass concentration (*dM_Dae_*) was calculated as follows:

(2) $$dM_{Dae}=dN_{Dae}\frac{\pi }{6}\;D_{ve}^{3}\rho_{P}$$

where Dae is aerodynamic diameter, *N* is number of particles, $D_{ve}$ is volumetric equivalent diameter, and $\rho_{P}$ is density of the particle. In this paper we set $\rho_{P}$ as 1.8 $g\;/{\mathrm{cm}}^{3}$ for fine PM2.5 and $\rho_{P}$ as 2.7 $g/{\mathrm{cm}}^{3}$ for coarse PM10 [16]. Figure 12 and Figure 13 are actual measurement data of PM2.5 and PM10 using TOF and β-ray equipment, respectively.

The distance between the two instruments is about 10 m. On 15 December 2022, from 3:00 to 5:00, the β-ray lost 2 h of data because the tape needed to be replaced manually. During the whole measurement period, the concentration value recorded by TOF is consistent with the measured value of β-ray, the mass concentration data of TOF is based on the assumption of the density of the analyzed particles, and there are some deviations from the average concentration data of β-ray. TOF equipment outputs data every 5 min, which is the average value within 5 min. β-ray outputs data once per hour, which is the average value within an hour. When the concentration of particulate matter in the aerosol fluctuates greatly within 1 h, the peak value of TOF data is higher, and it can reflect the real concentration at that time more correctly. This is because TOF equipment can measure the concentration of particles every 5 min, providing more accurate and real-time data than the β-ray equipment, which provides data once per hour. It is acceptable to have some deviations when compared to a β-ray instrument. Even in heavily polluted conditions, TOF instruments can accurately reflect the true picture of pollutants.

### 3.5. Performance Comparison

After the above comparative experiments and actual measurements, the feasibility of the method for measuring particle size proposed in this paper is confirmed. The method described in this article has many advantages over other types of equipment. The following Table 4 compares our equipment with other equipment mentioned in this article.

Compared to other methods, the TOF method has many advantages, such as that the TOF method can measure particles of varying sizes and masses simultaneously. It is also unaffected by water vapor, making it ideal for measuring particles in humid conditions. Additionally, the TOF method offers high measurement accuracy and real-time performance, and does not require any consumables.

Compared with TSI3321, our equipment can measure forward scattering light and backward scattering light at the same time. The addition of forward scattering light and backward scattering light increases the measurement sensitivity, and the ratio of forward to backward scattering light can further enrich the information of particles. Using digital acquisition technology and digital signal processing technology, various complex algorithms can be designed for high-concentration overlapping events to achieve a wide measurement dynamic range, and high concentrations can be measured. Additionally, digital signal acquisition provides for the establishment of a particle model library, which can be used to develop new applications. This combination of technologies allows for the acquisition of more accurate and detailed data on the particles in a sample.

## 4. Conclusions

Through verification, the method proposed in this paper can increase the minimum resolution particle size of TOF from 0.5 μm to 0.3 μm by employing 2 APDs to receive forward-scattered light and side-scattered light, and the ratio of forward scattering light to side scattering light can be used to further obtain the size and shape information of the particles.

The experiment for high concentrations shows that the anti-overlap algorithm proposed in this paper can effectively improve the applicability of the aerodynamic particle size spectrometer for high-concentration conditions. Based on the anti-overlap algorithm, our instrument can work at concentrations up to 8000 particles/cm^3^, much higher than the 1000 particles/cm^3^ of APS3321 [32].

The experiment for ambient aerosols shows the 5-day data of the comparison between the TOF instrument and the β-ray instrument to measure the ambient air. It can be seen that the concentration data of the two instruments are consistent. Since the mass concentration data of TOF is based on the assumption of the density of the analyzed particles, there are some deviations from average concentration data of β-ray.

It is concluded that the two APD methods used in this paper can improve the sensitivity of the instrument, and the anti-overlap algorithm based on the digital method can increase the upper limit of the instrument’s detection concentration. It laid the foundation for the development of a new generation of aerodynamic particle size spectrometer.

## Figures and Tables

**Table 1:** Methods for measuring particulate concentration and size.

Method	Advantage	Disadvantage
β-ray absorption	Good mass sensitivity.	Time consuming and expensive. Sampling paper tape replaced manually. Data output period is long (1 data/1 h). Measuring different particles sizes need cutting head.
QCM (Quartz crystal microbalance)	Good mass sensitivity.	Cannot measure particle size. Highly affected by water vapor. Measuring different particles sizes needs cutting head.
Charge transfer	System is simple. Mainly used for engine exhaust nanoparticle size detection.	Influenced by factors such as particle size changes, composition changes, and water vapor.
Optical scattering (Laser scattering)	Low cost. Accurate in low concentration. Suitable for large particle.	Inaccurate mass concentration. Concentrations will be underestimated because of overlapping particles.
Time of flight (Only have APS3321)	Suitable for 0.5–20 μm. High measurement accuracy. Measure mass concentration at the same time. High resolution, good stability. Unaffected by water vapor.	Be interfered with by overlapping particles in high concentrations due to defects of its signal processing circuit. Very expensive. High technical complexity.

**Table 2:** Particle properties in nozzle.

Particle Diameter (μm)	Relative Velocity (cm/s)	Particle Reynolds Number
0.5	40.0	0.01
1.0	1750.0	1.16
3.0	6490.0	12.90
10.0	10,600.0	69.60
15.0	11,500.0	114.00
20.0	12,300.0	163.00

**Table 3:** Standard deviation of TOF and Intensity of PM0.8\PM2.2\PM4.

Particle Matter	Std of TOF	Total Intensity of Scattered Light			Ratio (F/B)	Std of Light Intensity	Std of Light Scattering Particle
		Sum	Backward	Forward			
0.8	0.08	179	110	69	0.62	35.72	0.16
2.2	0.13	553	312	241	0.77	56.41	0.22
4.0	0.17	946	496	450	0.91	88.35	0.37

**Table 4:** Performance comparison of various instruments and equipment.

Instruments	Method	Measure (Particles Size)			Measure (Mass)	
		Range	Numbers	Resolution	Range	Resolution
Thermo Model 5014i ^1^	β-ray				Indirect ~10 mg/m^3^	0.1 μg/m^3^
QCM200 ^2^	QCM				Direct ~10 mg/m^3^	0.2 ng/m^3^
TSI 3091 ^3^	Charge transfer	5.6~560 nm	~10^7^ p/cm^3^	4 nm @56 nm	Indirect	10 ng/m^3^
TSI 8533 or 8534 ^4^	laser scattering	0.1~15 μm	~50,000 p/cm^3^	1.57 μm @2.2 μm	Indirect ~150 mg/m^3^	1 μg/m^3^
GRIMM model 1.107 ^5^	laser scattering	0.25~32 μm	~2000 p/cm^3^	0.5 μm @2.2 μm	Indirect	0.4 μg/m^3^
TSI3321 ^6^	TOF and laser scattering	0.5~20 μm	~1000 p/cm^3^	0.15 μm @2.2 μm	Indirect	0.1 μg/m^3^
Our equipment ^7^	TOF and laser scattering	0.3~20 μm	~8000 p/cm^3^	0.13 μm @2.2 μm	Indirect	0.1 μg/m^3^

^1^ Model 5014i [33]; ^2^ QCM200 [9]; ^3^ TSI3091 [34]; ^4^ Xiaoliang Wang’s Instrument [17]; ^5^ Hans Grimm’s Instrument [18]; ^6^ TSI3321 [14,15,16,35]; ^7^ TOF range can be extended to 0.3~40 μm.

## Data Availability

Data is contained within the article.
